# Why we should have more collaboration on HTA in Europe: the example of sofosbuvir

**DOI:** 10.1186/2052-3211-8-S1-O13

**Published:** 2015-10-05

**Authors:** Hedi Schelleman, Rudy Dupree, Finn B Kristensen, Wim Goettsch

**Affiliations:** 1 National health Care Institute, Diemen Zuid, 1112 XH, the Netherlands; 2 University of Southern Denmark, Copenhagen, 1399, Denmark

## Problem statement

Sofosbuvir is the first of a series of new and promising agents that can be used to treat chronic hepatitis C in adults. But even after price negations, the impact of sofosbuvir on health care budgets is too high to treat all affected patients in Europe.

## Objectives

To demonstrate, using sofosbuvir as an example, that separate national health technology assessments (HTAs) may not support the timely and consistent exchange of information and therefore joint HTAs may have additional value for timely and consistent decision-making around Europe. This may reduce obstacles to initiate voluntary joint price negotiations.

## Methodology

Study design: Review

Time period:1 August-1 November 2014

Setting: The study addresses the out-patient sector and examines the public sector.

Intervention: We sent a questionnaire in August/September 2014 and reviewed full HTA reports from September-November 2014.

## Results

The results of the questionnaire showed that, 7 months after sofosbuvir received market authorisation, in 11 countries the assessment of the effectiveness sofosbuvir had not yet started, in nine it was ongoing and in seven it was completed. Of the 11 countries that had not started an assessment, five Eastern European countries reported that the MAH had not yet submitted an application for reimbursement of sofosbuvir. The analysis also showed that in the seven reports from different European HTA organisations several relative effectiveness assessment elements were in common.

## Conclusions

At present, the MAH seems to set the pace of HTA assessments in Europe which reduced the possibilities for payers to participate in voluntary joint price negotiations. Furthermore, HTA organisations in Europe agree on key methodological aspects of their relative effective assessments, which supports the conclusion that joint relative effective assessment is feasible.

## Lessons learned and success factors

We assert that joint assessments may assist European countries in an earlier and more synchronized start in their discussion with the MAH on reimbursement and pricing of these new drugs. Crucial success factors for joint assessments are timeliness and topic selection.

